# Development and evaluation of a youth mental health community awareness campaign – The Compass Strategy

**DOI:** 10.1186/1471-2458-6-215

**Published:** 2006-08-22

**Authors:** Annemarie Wright, Patrick D McGorry, Meredith G Harris, Anthony F Jorm, Kerryn Pennell

**Affiliations:** 1 ORYGEN Research Centre, University of Melbourne, Locked Bag 10, Parkville, Victoria, Australia; 2 School of Population Health, University of Queensland, Locked Bag 500, Richlands, Queensland, Australia

## Abstract

**Background:**

Early detection and treatment of mental disorders in adolescents and young adults can lead to better health outcomes. Mental health literacy is a key to early recognition and help seeking. Whilst a number of population health initiatives have attempted to improve mental health literacy, none to date have specifically targeted young people nor have they applied the rigorous standards of population health models now accepted as best practice in other health areas. This paper describes the outcomes from the application of a health promotion model to the development, implementation and evaluation of a community awareness campaign designed to improve mental health literacy and early help seeking amongst young people.

**Method:**

The Compass Strategy was implemented in the western metropolitan Melbourne and Barwon regions of Victoria, Australia. The Precede-Proceed Model guided the population assessment, campaign strategy development and evaluation. The campaign included the use of multimedia, a website, and an information telephone service. Multiple levels of evaluation were conducted. This included a cross-sectional telephone survey of mental health literacy undertaken before and after 14 months of the campaign using a quasi-experimental design. Randomly selected independent samples of 600 young people aged 12–25 years from the experimental region and another 600 from a comparison region were interviewed at each time point. A series of binary logistic regression analyses were used to measure the association between a range of campaign outcome variables and the predictor variables of region and time.

**Results:**

The program was judged to have an impact on the following variables, as indicated by significant region-by-time interaction effects (p < 0.05): awareness of mental health campaigns, self-identified depression, help for depression sought in the previous year, correct estimate of prevalence of mental health problems, increased awareness of suicide risk, and a reduction in perceived barriers to help seeking. These effects may be underestimated because media distribution error resulted in a small amount of print material "leaking" into the comparison region.

**Conclusion:**

We believe this is the first study to apply the rigorous standards of a health promotion model including the use of a control region to a mental health population intervention. The program achieved many of its aims despite the relatively short duration and moderate intensity of the campaign.

## Background

The mental health literacy of Australian and international populations has been closely examined over the past ten years [1-4]. It is an important litmus test of a population's capacity to recognize mental health problems and seek appropriate help for them. Indeed mental health literacy is a key to promoting early detection and treatment of mental health problems and thus improving longer term outcomes [1,5]. Whilst a number of population health initiatives have attempted to improve mental health literacy with the aim of improving recognition and help seeking and reducing stigma [6-9], none to date have rigorously applied models to guide their development, implementation and evaluation. This is despite such standards being commonplace in other population health areas targeting physical health problems[10,11].

In this paper we describe the use of the Precede-Proceed Health Promotion Planning Model [12] which guided the development of a program designed to improve early identification of mood disorders and psychosis in young people. Most mental disorders typically first present during adolescence and young adulthood and these are often characterized by co-morbidity [13,14]. These specific disorders and age group were considered an important target for improving mental health as early detection and treatment at the time of first onset has been found to improve long-term outcome [15,16] and reduce the risk of future episodes of illness [17,18]. The program, The Compass Strategy, was a mental health literacy community awareness campaign targeting young people aged 12–25 in the western metropolitan Melbourne and Barwon regions of Victoria, Australia. The program was implemented from May 2001 to May 2003. Three key aspects of the program are outlined here – the population assessment, the campaign strategy and the evaluation results.

## Method

### Model for program planning

The Precede-Proceed Model [12] was selected to guide the development, implementation and evaluation of the program as it is a theoretically robust model that addresses all these phases of program development. As reviewed by Green and Kreuter [10] it has served as a successful model in several rigorously evaluated clinical and field trials, has been widely used by government and specialist health organizations (e.g. Centres for Disease Control and Prevention (CDC)), and has been successfully applied across a range of preventive health promotion programs including early detection initiatives. Its only other application in mental health has been to promote medication compliance amongst people with chronic mental disorders [19].

**Figure 1:**
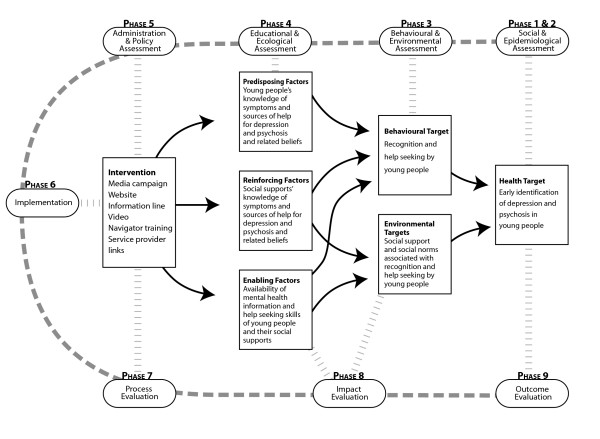
Application of the Precede-Proceed Model to The Compass Strategy.

The model involves nine phases (see Figure 1) and is based on the premise that a thorough assessment (*Precede*) should be made before planning a health promotion intervention, and evaluation (*Proceed*) is built in to the process to enable measurement of the effectiveness of interventions. Priority targets for intervention are established through each phase of the assessment process (phases 1–5) on the basis of causal importance in the chain of health determinants, their prevalence and their changeability. The results of this assessment process guide the development of the intervention (phase 6). The evaluation (phases 7–9) then tracks the impact of the intervention on factors identified as important targets in the assessment process.

### Setting

The adjacent Victorian regions of western metropolitan Melbourne (population 794,922) and Barwon (population 221,846), were selected for the intervention as they provided the geographic and demographic variability needed to evaluate the generalisability of an initiative such as this to metropolitan, regional and rural settings. This selection was also guided by the capacity of the regions' service system to respond to the needs of young people with mental health problems via specialist youth mental health services and recent General Practitioners' training initiatives in youth mental health.

The metropolitan region of south east outer Melbourne (population 536,718), and the adjoining regional/rural area of Mornington Peninsula and west Gippsland (population 154,697) were selected as a comparison region for the impact evaluation. Selection was based upon similarities in geographic and socio-demographic factors [20] as well as suitable distance from the experimental area to ensure the presence of a buffer zone should the intervention "leak" beyond the experimental region's boundaries.

### Population data sources

#### Focus groups

Four focus groups [21] with young people and parents affected by mental health problems were conducted in early 2001. These were based on focus group theory espoused by Krueger [22]. The aim was to explore ideas about how to convey messages regarding recognition and early help-seeking to young people and the rest of the public.

#### Service provider consultations

Approximately 30 consultation forums and presentations, with an average of 9 participants per session, were conducted prior to the campaign with workers from a range of key service sectors. Consultations included an overview of the rationale and strategies to be used by Compass, and an exploration of challenges faced by young people and service providers in recognising mental health problems and finding appropriate help.

#### Telephone survey

A cross-sectional telephone survey using structured interviews was conducted before the campaign [5] with a randomly selected sample of 600 young people aged 12–25 years from the experimental region and another 600 from the comparison region. Participants were presented with vignettes of a person with either depression or psychosis followed by questions related to recognition of disorder, best forms of treatment, perceived outcomes and exposure to the disorder and related media. The survey was repeated with an independent sample of 600 young people from each of these same regions 14 months after the campaign commenced.

### Assessment

#### Phases 1 & 2 – Epidemiological and social assessment

Typically the application of the Precede-Proceed Model commences in phase 1 with the examination of quality of life factors that are of prime importance to the community followed by an examination of health issues connected to the attainment or deprivation of these quality of life factors (phase 2). However, as mental disorders account for 55% of the total injury and disease burden in young Australians aged 15 to 24 years [23] increasing rates of treatment has the potential to substantially reduce disease burden and consequently improve health-related quality of life [24]. Therefore, further identification of health and social targets was not pursued, and the health target was identified as early identification and treatment of depression and psychosis in young people.

However an outstanding issue that usually occurs as part of the social assessment process is engagement of the target population and/or key organizational partners [12], which is vital to the long term success and relevance of an intervention. This sub-assessment required the identification of the key stakeholders likely to have an investment in the mental health of young people, the young people and families themselves who have been affected or may be in the future, and those organizations and services which take responsibility for the treatment of these young people. The stakeholders identified included:

• mental health services

• general practitioners

• community health services

• young consumers of mental health services or their family members

• the education sector

• youth and welfare services

• youth and mental health research programs

• mental health peak bodies

• local, State and Commonwealth government departments.

Elements of the social reconnaissance method [25] were used to determine relevant aspects of the social and cultural context of the community using community leaders (general, local and specialized) as informants or interviewees. A Project Development Group comprised of representatives from the above stakeholder groups was established to guide the process of reviewing relevant research, identify community leaders, representatives or groups who would be key informants and how they might best be approached, then review the interview results and guide further developments. The principal method used to access key informants was a series of service provider forums as well as consultations with key groups via established service provider meetings.

#### Phase 3 – Behavioural and environmental assessment

The focus of this assessment phase was the identification of the etiological factors, or determinants of health, in the behavioural patterns and environment of the population. Factors of particular interest were those associated with early identification and receipt of treatment for mental health problems.

##### Behavioural assessment

The behavioural assessment required a systematic analysis of behavioural links to the identified health target of early identification and treatment. A study of pathways to care [26] and results from focus groups suggest four overall steps in the process of early identification and entry into treatment: 1. recognition of the problem; 2. seeking help for the problem; 3. delivery of appropriate treatment and 4. compliance with treatment. The ranking of importance of these problems required an examination of how frequently they occur and whether they are clearly and potently linked to the health problem.

Recognition of depression, and especially psychosis, has been identified as quite limited in a number of studies [5,27] and is an essential step in the process of effective help seeking [1,5]. Help seeking at the earliest possible stage, in turn, is essential to early receipt of treatment [26]. However, rates of help seeking are poor, particularly in relation to mood disorders [28,29]. Indeed, recognition and help seeking were considered particularly worthwhile targets as previous population health interventions have demonstrated some success in increasing them [7], although this occurred over a four to five year period. Furthermore, recognition is likely to change more quickly than help seeking, as the former is knowledge based and the latter requires behaviour change [30].

Delivery of appropriate treatment and the promotion of treatment compliance were not considered priority targets as strategies targeting these issues were already being conducted in the target area by other health and mental health services. Instead liaison with these initiatives to maximize synergies was facilitated through their membership in the Project Development Group.

##### Environmental assessment

The environmental assessment process focuses on factors in the immediate social and physical environment that could be causally linked to the behavioural targets (Figure 1) or directly to the outcome of interest – viz. early identification and treatment. Social environment factors associated with recognition and help seeking (as revealed through focus groups, telephone survey and a previous study [31]) include social support for help seeking, social norms of expected behaviours and responses to mental health problems, contact with a social network, and the stigma associated with mental illness. Health care environmental factors, including accessibility and availability of services [28] and the quality of treatment by treating professionals [32,33], are important factors but were managed by other health and mental health services.

Social support and social norms for recognition and help seeking were determined to be the most worthy environmental targets, as strategies targeting them have previously demonstrated some success [6,7].

#### Phase 4 – Educational and ecological assessment

The focus in this phase is on the factors influencing health-related behaviour and conditions of living that require change in order for behavioural and environmental change to occur. The primary premise is that for behaviour change to occur one needs the cognitive and affective building blocks that provide the motivation to act (predisposing factors) as well as resources (enabling factors) and rewards (reinforcing factors).

##### Predisposing factors

Predisposing factors are the knowledge, attitudes, beliefs, values, perceived needs and abilities related to the motivation to act. The principal predisposing factors challenging young people (as revealed through focus groups, telephone survey and a previous study [31]) were limited knowledge and awareness of signs, symptoms and potential seriousness of depression or psychosis and treatments available, beliefs that they weren't susceptible to these disorders or that they would be stigmatized if they sought help, and a fear of being "locked up" in a psychiatric hospital.

##### Reinforcing factors

The prime sources of reinforcement identified by young people (as revealed through focus groups, telephone survey and a previous study [31]) in the form of positive feedback and social support for recognition and help seeking were family and friends followed by teachers, counselors, and lay leaders, for example church leaders. Indeed, in many instances young people in the focus groups said they needed those around them to recognize that they were experiencing a mental health problem as they couldn't at the time of onset.

Focus groups and service provider consultations revealed that these social supports faced the same challenges as young people, that is, knowing the signs and symptoms and sources of help, and a lack of belief in the susceptibility to the disorders.

##### Enabling factors

In regard to enabling factors that facilitate the process of recognition and help seeking, accessibility and availability of quality services are obvious factors and these concerns were raised in service provider consultations. However, as highlighted earlier, these issues were managed concurrently by other programs. Lack of information about mental health problems and appropriate sources of help have been identified as a barrier to effective help seeking by young people and parents (as revealed through focus groups). The development of related skills could further enable recognition and help seeking[12] although this was determined to be a secondary target to other factors already identified that are necessary pre-requisites to skill attainment.

Therefore, the priority target factors identified in this phase for both young people and their social supports was the provision of information to improve knowledge of symptoms and sources of help for depression and psychosis, and related beliefs regarding the severity of these problems, and young people's susceptibility to them.

#### Phase 5 – Administration and policy assessment

This phase requires an analysis of the policies, resources, and circumstances in an organizational setting and context that either hinder or facilitate the development of a health promotion program. To a large extent this assessment was intrinsic to the establishment of the project. Funding was made available for grant application in the context of the new Australian Government mental health policy focusing on early intervention and mental health promotion [34], which meant that it was likely to complement other initiatives borne of this policy. Furthermore, provision of funding was based on the demonstration of support and capacity in the community and the service system for an initiative such as this.

### Intervention

#### Phase 6 – Implementation

In this phase the predisposing, enabling, and reinforcing factors that influence behaviour conducive to recognition and early help seeking became the factors that shaped the program. The development and implementation of the intervention occurred over a three year period and was guided by the Process Model of Social Marketing Program Development [35]. The campaign itself ran from May 2001 to May 2003.

##### Strategy development

The development of the overall strategy was undertaken largely through ongoing consultation with the Project Development Group. This helped to ensure that the intervention complemented related mental health promotion programs and facilitated liaison with existing community programs and organizations so that they could assist in the delivery and promotion of the project. As the program progressed, the Project Development Group also provided a means of feedback about how the campaign was being received in the community and suggestions for refinement.

The nature of the target region was also examined to determine opportunities and limitations for health promotion activities. For example, as the intervention was restricted to one geographic area, and a matched area was studied as a comparison region, strategies such as television advertising could not be used as, due to local television network coverage, they would broadcast beyond the target region and risk contaminating the comparison region.

##### Audience segmentation

Whilst young people and their social supports were identified as key targets during phase 3 of the assessment process, in practical terms the target audience was divided into adults (mainly parents), and young people (self, friend or family member). Young people were further segmented according to socio-economic status and gender. Socio-economic disadvantage and lower literacy have been associated with poorer response to media campaigns, with personalized outreach approaches likely to be more effective [36]. Hence this group required a different approach to minimize the likelihood of increasing health inequalities. Some segmentation according to gender was also undertaken due to lower help-seeking rates in males [37] and higher prevalence of depression in females[38].

##### Development of key messages

The key messages evolved during the preliminary phase of the campaign and were gradually refined into a core slogan of "get on top of it before it gets on top of you" using a common phrase to bring to life the benefits of early recognition and help seeking. Based on the identified predisposing and reinforcing factors (Figure 1) and the Health Belief Model [39] as it relates to help seeking, this core message was supplemented by five other key messages – that young people are particularly susceptible to mental health problems, that symptoms should be taken seriously, a listing of core symptoms, getting help early is a key to successful treatment and directions to sources of more information (a website and an information line). The message was delivered in gender neutral "third person" so it would be relevant for those potentially affected or those close to them.

##### Channels of communication

According to Green and Kreuter [12], direct communications to the target population strengthen predisposing factors, indirect communications to key people in social networks strengthen reinforcing factors and training strengthens enabling factors. The selection of channels of communication (see Table 1) was determined by focus group feedback and national survey findings [31] with an emphasis on the use of multiple settings and strategies to maximize the likelihood of the message being received[40].

A range of major and minor media were reviewed for their capacity to reach young people and/or parents and their capacity to convey the core messages (see additional files 1, 2, 3, 4 for examples of Compass media campaign material). Media dissemination, whilst at times costly, provides an umbrella for attention to the issue, is able to reach the general population, and assures control over and adds authority to the core messages. It also allows for agents of reinforcement (peers, parents, schools etc.) to be exposed to the same message and therefore potentially support the desired behaviour [41]. The website and information line modules were designed to provide more information related to the core campaign messages, and to address the target enabling factors of skills in recognition and help seeking. The video and Navigator modules were developed for their potential to address all predisposing, enabling and reinforcing factors.

The selection of interventions was also guided by the Stages of Change Model [42] and stages of innovation adoption [36]. Both models have been widely applied to the field of population health [11]. The primary premise in applying these models is that different people will be at different stages of readiness to contemplate mental health issues and the benefits of early help seeking, and will move through these stages at varying rates as part of the process of recognition and help seeking. The overall modular strategy was designed to facilitate progress through these stages.

##### Pre-testing

Where possible, all elements of the major and minor media were pre-tested with the target audience of young people and adults using previously established health campaign pre-testing methods [43]. The aim was to ascertain message comprehension, appropriateness to youth culture, reading level, user friendliness, accuracy of take out message, capacity to engage the target audience as well as to determine any potential negative effects. The website was pre-tested for functionality and ease of navigation, and the information line underwent a series of "dry runs" prior to commencement. The video module was developed in consultation with the target audience of potential users, mainly education departments and secondary school teachers, to test out concepts and ensure synergy with current curricula and school policies. Navigator was piloted with five groups, refined and then implemented in full.

##### Implementation strategy

Each module had its own implementation plan to ensure appropriate timing of campaign modules in relation to stage of readiness for change and to ensure effective targeting of high impact periods e.g. for major media this was school holidays and for minor media, service providers and Navigator this was school term. The result was an overall implementation plan that proceeded in waves of intensity with a varying number of modules implemented at any one time. This strategy is common in the commercial market place and is particularly useful in making sure the impact of media strategies isn't reduced through overuse.

### Ethical approval

Ethical approval for the focus groups was obtained from North Western Mental Health Behavioural and Psychiatric Research and Ethics Committee and from Barwon Health Research and Ethics Advisory Committee. Focus group participants gave written consent for their participation after reading a plain language statement describing the study.

Ethical approval for the telephone survey was obtained from the Ethics Committee of the Victorian Government Department of Human Services. Survey participants gave oral consent to participation after being read a plain language statement describing the study by the interviewer, and for those aged under eighteen years of age, oral consent was obtained from a parent or guardian.

## Results

### Evaluation

#### Phases 7, 8 & 9 – Process, impact and outcome evaluation

The evaluation process was developed concurrently with the intervention. The primary goal was to track whether the program was being implemented effectively and whether it was impacting on the targets set during the assessment process (Figure 1). The evaluation structure was guided by that outlined as part of the Precede-Proceed Model [12] and the framework developed by Hawe and colleagues [44]. The development of the evaluation plan and review of its implementation was guided by an Evaluation Committee comprised of experts in the field of mental health literacy, qualitative and quantitative research methodologies and population health program evaluation. The evaluation results described here are for the first 14 months of the program, and don't include the Navigator and Video modules which were implemented later.

##### Process evaluation

Process evaluation (Table 2) examined whether the program was being implemented as planned and operating effectively, and allowed for adjustments to be made to the strategy as problems or deficits arose. Regular reviews of the attainment of key performance indicators (KPIs), that is, the pre-defined essential activities linked to the attainment of established program goals in the strategic plan, were undertaken by the Project Development Group. Sixty-five KPIs were developed to measure the achievement of the program objectives, including the development of a multimedia community awareness campaign for young people, their families and community leaders and a complementary school-based awareness campaign, as well as the development of an evaluation strategy to measure campaign awareness and mental health literacy. These objectives were then linked to the goals of increased mental health literacy and earlier help seeking identified in phases 3 and 4.

Determining whether the target population was being reached was measured in a number of ways. A "pop-up" questionnaire on the home page of the website enabled the measurement of the source of referral to the website (e.g. cinema advertisement, newspaper, school), the age and gender of the user and their postcode. A similar questionnaire was used as part of the information line. This was supplemented by monitoring the demand for minor media resources (e.g. brochures, postcards, posters) by schools and community agencies. Feedback from these services and from website users helped measure the utility of these resources.

The effectiveness of the information line and website were tracked by monitoring hits to the website and calls to the information line. Examination of these data in parallel with the varying intensity of the campaign waves indicated that as the campaign intensity increased so did visits to the website and calls to the information line.

##### Impact evaluation

The telephone survey was the primary measure for impact evaluation. The baseline survey was repeated with an independent sample of 600 young people in the experimental region and 600 young people from the comparison region. The sample was stratified by age (12–14 years (25%), 15–17 years (25%) and 18–25 years (50%)) and region at both time points. Because there was no existing research to indicate a likely effect size, a power analysis was carried out using Cohen's [45] "small" effect size as the criterion (i.e. h = 0.2 for a difference between independent proportions). For this effect size, a sample of 392 in each region gave 80% power with an alpha of 0.05, and a sample of 584 had the same power with an alpha of 0.01.

The pre-campaign study response rate was 89.7% and the post-campaign response rate was 86.8%. Logistic regression analysis was used to ascertain whether a range of sample characteristics varied across time points or regions. There were no significant region-by-time interaction effects for sample differences in gender, school enrolment, unemployment or language other than English spoken at home. Age was not included in this analysis due to sample stratification.

A series of binary logistic regression analyses were used to measure the association between a range of campaign outcome variables and the predictor variables of region (experimental, comparison) and time (pre-campaign, post-campaign). The regression analysis was carried out controlling for vignette where outcome variables specifically related to the depression or psychosis vignette. The outcome variables considered in the analysis were correct recognition of disorder, form of help recommended, helpfulness rating of professionals and medications, perceived prognosis, perceived likelihood of discrimination, exposure to mental illness in family and friends, self-identified depression, help sought for the first time for self-identified depression during the time of the campaign, likely behaviours associated with the disorder (e.g. suicide), barriers to help seeking for self, correct estimate of prevalence and exposure to mental health media campaigns. The program was judged to have an impact if there were significant region-by-time interaction effects. Results using Wald statistics are reported in Table 3.

Significant results were observed for the outcome variables of perceived suicide risk, barriers to help seeking ("what others might think" and "thinking that nothing can help"), media exposure, correct prevalence estimate, and self-identified depression. There was an overall significant change in the proportion of those seeking help over time in the experimental region. However, when the help-seeking rate was examined specifically for those identifying themselves as having a mental health problem, the effect was non-significant. Non-significant effects were recorded for all other outcome variables including the key targets of recognition and knowledge of sources of help. A post hoc analysis revealed a greater impact of the campaign on the same outcome variables (Table 3) in the Barwon sub-region.

##### Outcome evaluation

For this level of evaluation, it was planned to examine changes in service utilisation, duration of untreated illness and number of contacts in pathways to care. However, practical difficulties in gathering data over the whole intervention region meant that this plan could not be achieved.

## Discussion

We believe this is the first study to apply the rigorous standards of a health promotion model to a mental health population intervention. The program achieved many of its aims despite the relative short duration and moderate intensity of the campaign.

Process evaluation revealed that the website was a far more frequently used source of information than the information line, and was more effective at attracting use by the target age group of young people. Newspapers, schools, posters and General Practitioners were the most effective website referral sources. Schools were also the greatest source of service provider demand for print materials.

Results of the impact evaluation telephone survey revealed significant changes in the target region across the range of impact targets. The overall recall of mental health campaigns was significantly higher in the target region. This suggests that the enabling factor objective of making mental health information more available was achieved. Three key predisposing and reinforcing factors showed significant change. Beliefs regarding the risk of suicide associated with depression and psychosis increased or stabilised in the target region whilst it declined in the comparison region. "Thinking that nothing can help" decreased as a barrier to help seeking in the target region, whilst it either increased or stabilised in the comparison region, suggesting that young people became more positive toward the potential benefits of treatment. Self-identified depression significantly increased in the experimental region and is reported here as a predisposing factor as it is most likely to be an indication of increased awareness of depression rather than an increase in prevalence per se [9]. This interpretation is supported by the finding that the relative proportion of these self-identifiers who had sought help did not change significantly over time.

In relation to the environmental targets of social supports and social norms, perceived negative evaluation by others regarding help seeking decreased in the experimental area, and perceived prevalence of mental health problems increased. A small but significant increase in the behavioural target of help seeking during the implementation of the campaign was observed in the target area.

The high response rate for the telephone surveys and the comparability of sample characteristics across time and regions strengthens the credibility of the results. The measurement tool itself has established validity [46], although its validity in telephone interview format has not been established. Observed changes may be an underestimate of the impact of the campaign given that a small amount of media distribution error resulted in some of the print material "leaking" into the comparison region and it was not possible to put geographical restriction on website access.

The use of website and information line referral data was a useful process evaluation tool, but several limitations must be considered. The use of visits per month may be an underestimate of individual actual visitors to the website as firewalls on computer networks within services or institutions will only represent this as one visitor when it may be several people from the same network. Also, the collection of referral source data was at times open to ambiguity where a referral source could be categorised under more than one heading e.g. poster, GP (poster in waiting room) or school (poster on premises). Similarly, referral from "school" to the website could be through recommendation from a school counsellor for personal use or research for a school project on mental health. Furthermore, the limitations of the referral source data as a measure of campaign effectiveness must be considered in relation to major and more expensive media such as radio and cinema advertising. Whilst neither of these media rated well as a referral source, it is difficult to ascertain the degree to which they had a role in priming or reinforcing key messages of the campaign. Indeed, the post hoc analysis revealed a greater impact of the campaign in the Barwon region and this was also the only experimental sub-region where radio advertising was implemented and there was a higher media distribution rate per head of population.

This is one of only a small number of studies that have used a comparison region to evaluate a mental health literacy community awareness campaign regarding depression [9,47], and the first study using this methodology in relation to psychosis. Data from the comparison region was critical in establishing the effectiveness of Compass as the strategy was operating concurrently with a range of national mental health community awareness initiatives, including *beyondblue: the national depression initiative *[48], MindMatters school based initiatives [49], and campaigns by SANE Australia [50].

In regard to time taken to have a significant impact on mental health literacy, comparisons are difficult due to differences in study design and campaign intensity and spread. Hegerl and colleagues [47] reported no significant impact after a 10 month regional campaign, Jorm and colleagues [9] reported significant changes over a nine year period which included a four year national campaign, whilst Compass demonstrated significant changes after a 14 month regional campaign.

Whilst a number of psychosis studies have examined the impact of a community awareness campaign on help seeking, in particular duration of untreated symptoms [51-53], they have not reported on the interim impact of these campaigns on mental health literacy. This may be of particular importance where a community awareness campaign does not demonstrate an impact on duration of untreated illness [52] or help seeking, as the campaign may not have had enough time to take effect. Timely measures of mental health literacy may provide evidence that some change has occurred, heralding longer term impacts on help seeking, so that campaigns can be continued. However, it must be acknowledged that evidence of this study's effects on help seeking have not been fully evaluated.

## Conclusion

The Compass Strategy was effective over a relatively short period of time with a moderate intensity campaign. This effectiveness may be attributed to the rigour of the Precede-Proceed Model which facilitated fine tuning of the campaign targets through the population assessment process, the use of evidence based campaign strategies, and the capacity to refine the campaign elements in response to regular reviews of process evaluation findings. Indeed, the Precede-Proceed Model may provide an efficient means for designing effective mental health community awareness campaigns in the future as it is potentially adaptable to all contexts and mental disorders.

The local media materials, website and video developed as part of Compass are currently being disseminated throughout the state of Victoria, Australia. Dissemination of Compass modules internationally would need to be considered in the context of appropriate fit with identified priority targets following the application of the Precede-Proceed Model to the population of interest.

## Competing interests

The author(s) declare that they have no competing interests.

## Authors' contributions

AW was the Project Manager and was responsible for conception and design of the study; funding acquisition; coordination of the intervention and data acquisition; analysis and interpretation of the data; and drafting of the manuscript. PDM was the Project Director and was involved in the conception, acquisition of funding, project design and management, interpretation of the data and production of the manuscript. MGH was involved in the design, coordination and evaluation of the project and drafting of the manuscript. AFJ was involved in the planning of the evaluation and in the analysis and writing of the paper. KP was involved in the acquisition of funding, design and coordination of the study, and drafting of the manuscript. All authors read and approved the final manuscript.

## Pre-publication history

The pre-publication history for this paper can be accessed here:



## Supplementary Material

Additional File 1 Compass Brochure Click here for file

Additional File 2 Example of Compass newspaper advertisement Click here for file

Additional File 3 Example of Compass Postcards Click here for file

Additional File 4 Example of Compass Poster Click here for file

## Figures and Tables

**Table 1:** Compass campaign modules

**Module**	**Stage**	**Resources and rationale**
Major media	Pre-contemplation and contemplation* Awareness and interest^▲^	***Cinema, radio, local newspaper, & youth magazine advertisements and newspaper editorials*** Provided high exposure and increased sense of authority to key messages
Local media	Pre-contemplation and contemplation* Awareness and interest^▲^	***Posters, brochures, postcards*** Passive application – Displayed in a wide range of local environments – health, welfare, youth, education, & recreation settings, local community events. Targeted environments where people were likely to be thinking about their own health and well-being or their connection to family and friends. Active application – Used in classroom, counselling or as a school newsletter supplement. Used as a reference to facilitate discussion or specifically referred to as useful reference material.
Website	Preparation* Trial^▲^	***Getontop Website*** Provided more detailed information about disorders. Help seeker tips and search engine for local sources of help.
GetOnTop Mental Health Info Line	Preparation* Trial^▲^	***Mental health advisors ***available Mon-Fri 10.30 am – 8.00 pm Provided personalized information about disorders and help-seeker coaching. ***Call Centre ***able to respond to all calls with basic information 24/7 or arrange advisor call back ***Information booklet ***was mailed or emailed on request. Designed to mirror the detailed info available on website
Video & facilitators manual	Pre-contemplation & contemplation* Awareness & interest^▲^	***Video ***scenarios depicting early signs of illness and strategies for help seeking to trigger discussion in the classroom or info sessions ***Facilitator manuals ***included suggestions for discussion topics, fact sheets and work sheets to be used along with the video
Navigator Program	Addresses all stages*^▲^	***Training workshops for lay professionals***, based on Mental Health First Aid Training [54] concepts, focused on knowledge and skill development in how to assist socio-economically disadvantaged and marginalised young people with early recognition and help seeking.
Service provider strategies	Action* Behaviour adoption^▲^	***Newsletters, visits and presentations*** Targeted specialist and generic mental health service providers and gatekeeper services as they are a key to reaching young people and those close to them. They need to be kept informed as key stakeholders in help seeking. Strategies focused on facilitating consultation, building strong links, and updating on resources and project progress.

*Stage of Change [40].

^▲ ^Stage of adoption [34].

**Table 2:** Process evaluation results

**Process evaluation measures**	**Results**
Attainment of Key Performance Indicators as outlined in Strategic Plan	93% of Key Performance Indicators achieved
Media distribution rates	• 7,630 cinema advertisements • 195 radio advertisements • 21 weeks of Youth Magazine advertisements • 15,000 post cards • 10 newspaper editorials • 15 newspaper advertisements (average circulation 20,000 per advertisement) • 600 posters to GP surgeries • 47 professional and service newsletter articles • posters and pamphlets to 320 agencies • Compass newsletter to 532 services • 20,000+ stickers distributed to all secondary schools in the target region
Website referral data	• Average of 465 visits per month • 66% of visitors female • 47.2% visitors aged 12–25; 31% aged 26–39 • Referral sources – newspaper (17%), school (12%), poster (11%), GP(11%), cinema (4%), radio (2.5%)
Infoline referral data	• Average of 28 calls per month • 64% of callers female • 24% callers aged 12–25; 41% aged 26–39 • Referral sources – pamphlet (26%), newspaper ad (17%), letterbox brochure (17%), newspaper/radio editorial (10%)
Demand for minor media	157 service provider requests for Compass materials • 46.4% secondary schools • 18.4% community health services • 7.6% mental health services

**Table 3:** Percentage of survey respondents reporting outcomes pre- and post-campaign in experimental and comparison regions.

Outcome	Experimental %		Comparison %		p value*	OR (CI)*
	Pre	Post	Pre	Post		
Correct recognition – depression	50.6	59.3	46.6	58.2	0.981	.99 (.71–1.39)
Correct recognition – psychosis	24.7	34.7	25.8	32.5		
						
Correct help identified – depression	55.8	48.6	49.3	46.9	0.906	.98 (.71–1.36)
Correct help identified – psychosis	62.0	61.6	63.4	57.1		
						
Helpfulness rating of professionals						
GP Helpful – depression	81.0	73.9	79.1	77.1	0.688	1.08 (.75–1.55)
GP Helpful – psychosis	69.8	68.8	69.9	66.9		
Psychiatrist Helpful – depression	71.3	75.0	76.4	74.0	0.387	.84 (.56–1.25)
Psychiatrist Helpful – psychosis	85.8	85.6	81.7	82.1		
Social Worker Helpful – depression	75.5	74.6	79.7	80.7	0.230	.79 (.54–1.16)
Social Worker Helpful – psychosis	77.3	77.8	76.4	76.0		
Psychologist Helpful – depression	69.7	73.2	72.6	71.6	0.288	.82 (.56–1.19)
Psychologist Helpful – psychosis	77.3	78.1	80.7	78.6		
						
Helpfulness rating of medications						
Antidepressants Helpful – depression	43.5	47.9	42.2	50.3	0.513	1.17 (.74–1.85)
Antipsychotics Helpful – psychosis	41.7	46.6	37.6	39.3	0.589	.88 (.56–1.39)
						
Correct prognosis – depression	31.6	33.2	30.7	37.3	0.542	.90 (.64–1.26)
Correct prognosis – psychosis	30.5	36.3	34.3	30.8		
						
Perceived likelihood of discrimination – depression	57.7	50.4	55.7	55.5	0.134	1.29 (.92–1.81)
Perceived likelihood of discrimination – psychosis	75.3	70.6	67.3	67.5		
						
Perceived suicide risk – depression	14.2	20.4	23.6	17.8	0.005	.57 (.38–.84)
Perceived suicide risk – psychosis	23.1	23.4	32.4	25.0		
						
Family or friends experienced illness – depression	43.5	51.1	47.0	52.1	0.921	.98 (.71–1.36)
Family or friends experienced illness – psychosis	34.6	38.1	32.4	37.7		
Family or friends received help – depression	32.3	39.3	34.5	39.4	0.970	.99 (.71–1.40)
Family or friends received help – psychosis	28.1	30.6	27.1	31.5		
						
Barriers to help						
Perceived negatively by treating person – depression	15.2	21.4	21.6	18.5	0.108	.72 (.48–1.07)
Perceived negatively by treating person – psychosis	22.4	20.3	23.9	20.5		
What others might think of you – depression	14.2	8.9	8.8	13.0	0.013	1.86 (1.13–3.06)
What others might think of you – psychosis	12.9	14.4	9.8	14.9		
Thinking that nothing can help – depression	6.5	3.2	3.4	3.1	0.04	2.33 (1.03–5.23)
Thinking that nothing can help – psychosis	5.1	3.4	3.6	5.8		
Concern about likely treatment – depression	2.3	3.6	2.7	2.4	0.308	.62 (.25–1.55)
Concern about likely treatment – psychosis	3.7	4.1	3.9	2.9		
Concern about likely diagnosis – depression	5.5	3.9	4.7	1.7	0.119	.53 (.24–1.18)
Concern about likely diagnosis – psychosis	3.4	5.3	5.9	4.2		
						
Media exposure	26.1	43.2	31.4	35.3	0.001	.55 (.39–.78)
						
Self-identified depression	26.0	34.0	30.4	30.3	0.029	.67 (.47–.96)
						
First sought help in previous year	3.8	5.7	6.3	4.2	0.022	.41 (.19–.88)
						
Correct prevalence estimate	20.0	24.7	29.1	22.8	0.002	.55 (.37–.80)

*Region-by-time interaction effects
