# Supplementary material for: Development and evaluation of a youth mental health community awareness campaign – The Compass Strategy
Source: BMC Public Health. 2006 Aug 22;6:215. doi: 10.1186/1471-2458-6-215 (PMC1564138; doi:10.1186/1471-2458-6-215)
Supplement: Additional File 1 — Compass Brochure [file 1471-2458-6-215-S1.pdf]

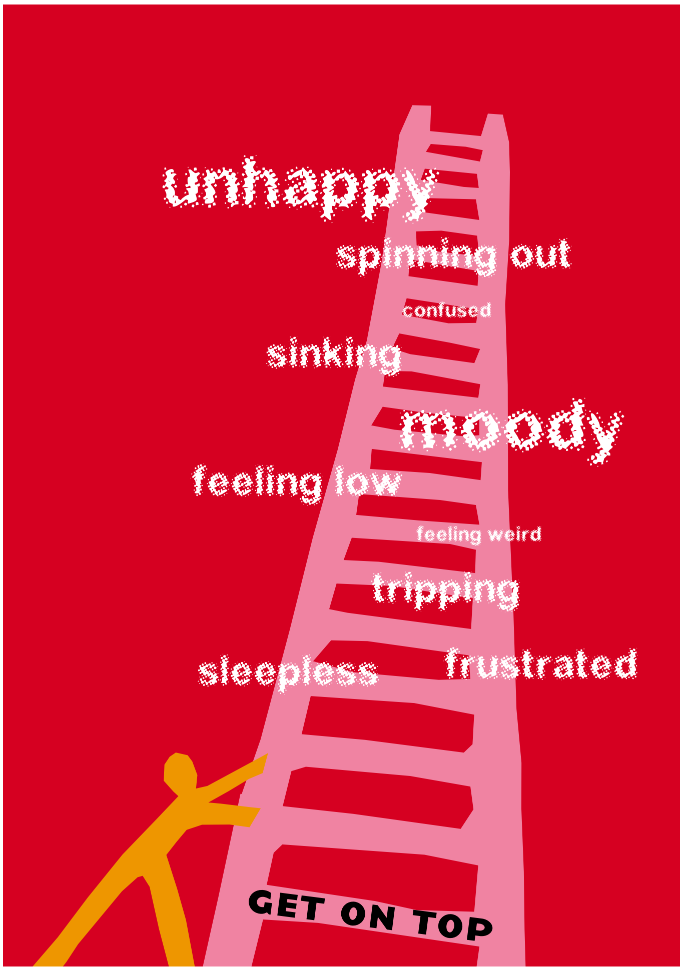

FACTS

> Every year **1 in 4 young Australians** aged 18-24 will experience a **mental health problem**. For those aged 12-16, the odds aren't much better – up to 1 in 5.

> **Depression**, and **Psychosis** are mental health problems that tend to first occur during adolescence and young adulthood.

> **Early detection** and treatment increases the likelihood of recovery and can prevent mental health problems from occurring in the future.

> **Help is available.** So get on top of it, before it gets on top of you.

Depression

Most people have felt low for brief periods of time. Feeling 'depressed', sad or unhappy is a normal reaction to experiences that are stressful, upsetting or difficult to come to terms with. The feeling might last for a few hours, a few days or even a week but then it goes away. But when it goes on and on, or when it is so bad that it dominates and starts interfering with someone's quality of life - it has become a serious medical problem, which requires attention. Depression may either start suddenly or build up gradually over days or weeks.

Symptoms

Depression affects every system of someone's body and can interfere with many aspects of their life.

Early signs of depression include difficulty coping with school or work, and feeling moody or irritable a lot of the time.

Later on, a person might experience more obvious symptoms. Typical symptoms are:

- feeling down or irritable most of the time
- loss of interest or pleasure in life
- feeling guilty, bad or worthless
- change in appetite or weight
- tiredness or lack of energy
- thoughts of death or dying
- sleeping problems
- difficulty concentrating or making decisions
- avoiding friends, fami;ly and regular activities.

If you know someone who has had five or more of these symptoms for two weeks or more it is quite possible they might be suffering from depression.

Psychosis

When people hear the word psychosis or psychotic, many think of violent images and people who are aggressive or out of control. This perception is incorrect. Psychosis is a medical condition that has nothing to do with violence. Psychosis describes a state of mind in which a person experiences a distortion of, or loss of contact with reality.

Symptoms

The very early signs of psychosis present as a combination of several subtle changes that are definitely different to how someone usually feels. These early signs include:

- Feeling depressed or sad most of the time
- Having difficulty relating to family and friends
- Having more difficulty coping with work or school
- Feeling very tired or lacking in energy
- Feeling paranoid or worried about other people and their actions
- Noticing a change in the way things look or sound
- Noticing things in the surroundings that other people don't.

Later on, as psychosis develops, a person may experience more obvious symptoms, such as:

- Confused thinking or difficulty making sense to others
- Strange or unusual ideas that aren't based on reality
- Seeing and hearing things that others can't
- Changed feelings or behaviour
- Behaving quite differently to usual.

Getting Help

Fortunately, effective treatments are available for depression and psychosis. The recommended treatments will depend on the symptoms, severity and the circumstances of the person. Getting help early is a key to successful treatment and there are many different types of people that can help, including:

General Practitioners (GP's)
Your local or family doctor.

Counsellors

Mental Health Services

You can visit our website
getontop.org
or call our information line
1300 73 22 11
to find the closest source of help in your area or just to find out more information.

© UNIVERSITY OF MELBOURNE 2001

> *Tessa's experience*

When Tessa became unwell with depression she felt as though no-one could like her. She began to wonder just what was the point of anything. At first she lost interest in everything and didn't feel like going out with her friends as much. She also found it difficult to concentrate on her work.

Over a couple of weeks she started to feel tired all the time and didn't sleep properly. She would wake up early before the rest of the family and lie there feeling deeply sad, all alone and forgotten by the world. She felt as though everything was darkness and full of nothing, stretching out in front of her forever with no chance of anything ever feeling better again. Thinking of this made her feel that dying would be better than living.

> *Ben's experience*

When Ben was becoming unwell with psychosis, no one really knew what was going on. He started to feel unhappy and worried. For some reason things around him seemed somehow changed and unusual.

Over time, he started to worry that something bad was going to happen to him. He began to withdraw from other people because he felt scared and suspicious of them, even though he didn't really know why.

He also noticed that his hearing and vision were different and at times he thought he could hear someone whispering to him, even though he was all alone.

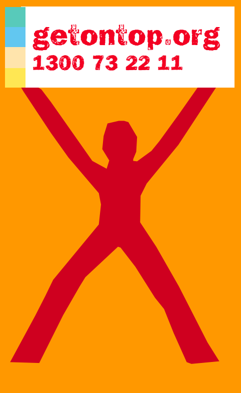

the compass strategy
get on top of it, before it gets on top of you
