# Supplementary material for: Development and evaluation of a youth mental health community awareness campaign – The Compass Strategy
Source: BMC Public Health. 2006 Aug 22;6:215. doi: 10.1186/1471-2458-6-215 (PMC1564138; doi:10.1186/1471-2458-6-215)
Supplement: Additional File 2 — Example of Compass newspaper advertisement [file 1471-2458-6-215-S2.pdf]

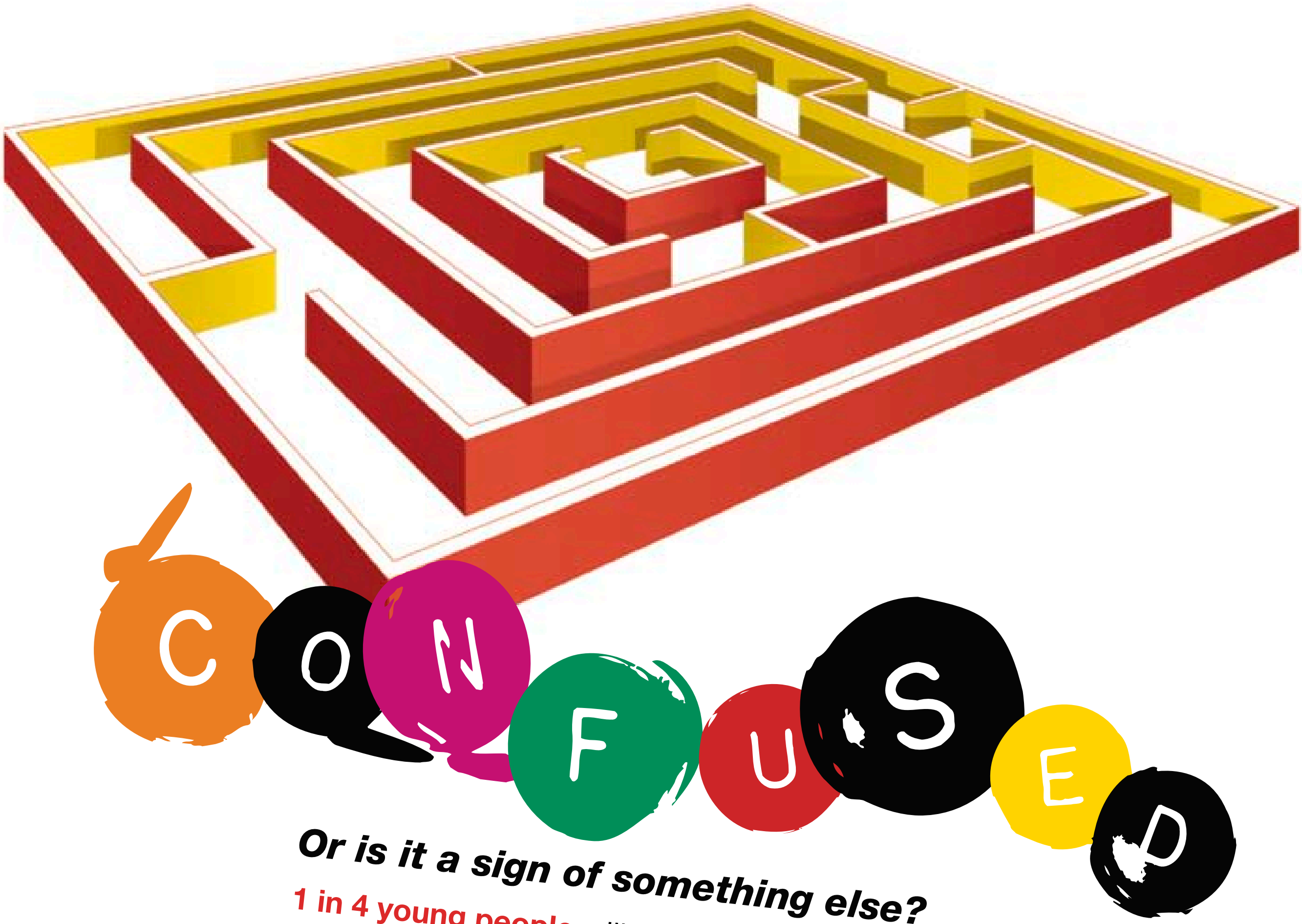

Or is it a sign of something else?

1 in 4 young people will experience a mental health problem, such as **psychosis.**

Signs include:

> Finding it more difficult than usual to cope with work or study
> Difficulty relating to family and friends
> Seeing and hearing things that others can't
> Strange or unusual ideas that aren't based on reality

Getting help early is a key to successful treatment.

To find out how you can help someone get on top of psychosis, call **1300 73 22 11** or visit **www.getontop.org**

COM025D - © UNIVERSITY OF MELBOURNE 2002

the compass strategy
get on top of it, before it gets on top of you

Example of Compass newspaper advertisement
