# Supplementary material for: Development and evaluation of a youth mental health community awareness campaign – The Compass Strategy
Source: BMC Public Health. 2006 Aug 22;6:215. doi: 10.1186/1471-2458-6-215 (PMC1564138; doi:10.1186/1471-2458-6-215)
Supplement: Additional File 3 — Example of Compass Postcards [file 1471-2458-6-215-S3.pdf]

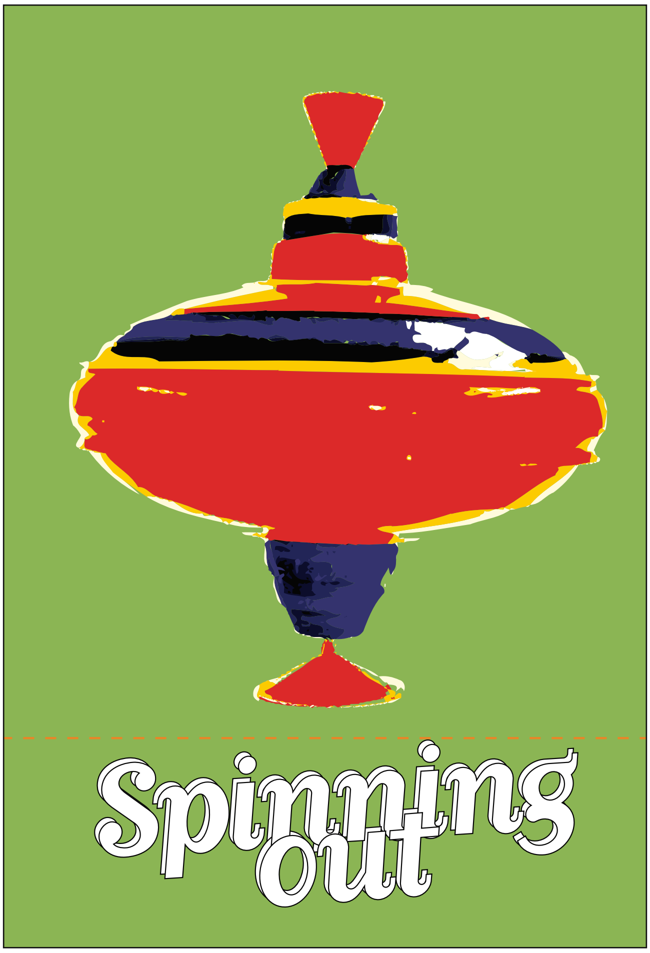

the compass strategy
get on top of it, before it gets on top of you

Finding it more difficult than usual to cope with work or study can be an **early sign of psychosis**.

Other signs include:
> Difficulty relating to family and friends
> Seeing and hearing things that others can't
> Strange or unusual ideas that aren't based on reality

1 in 4 young people will experience a mental health problem, such as psychosis.

Getting help early is a key to successful treatment.

To get on top of depression and psychosis call
1300 73 22 11
or visit **www.getontop.org**

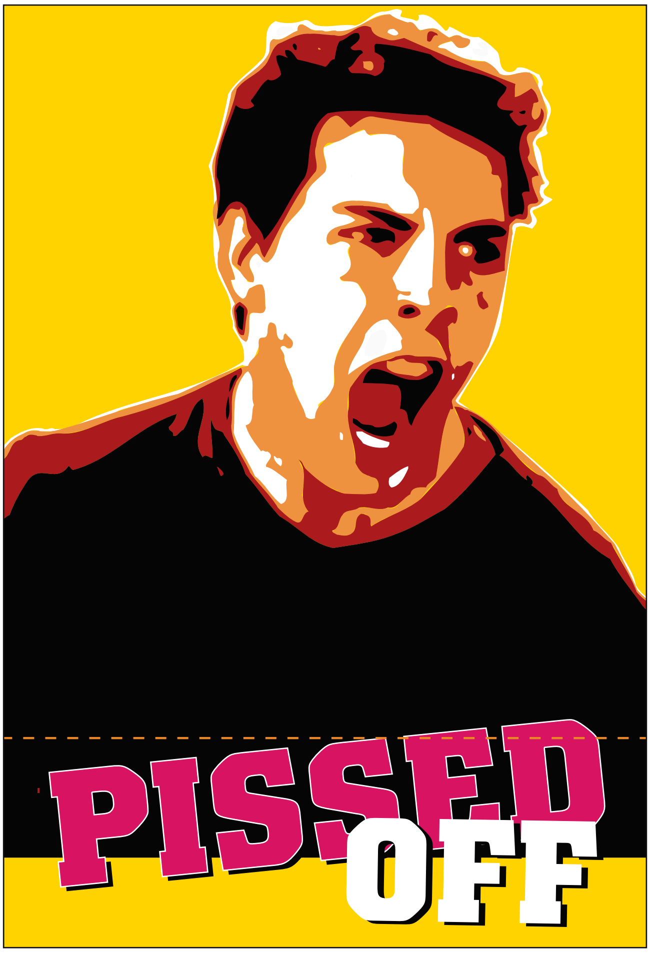

the compass strategy
get on top of it, before it gets on top of you

Feeling irritable most of the time can be an **early sign of depression**.

Other signs include:
> Feeling down most of the time
> Loss of interest or pleasure in life
> Difficulty coping with study or work

1 in 4 young people will experience a mental health problem, such as depression.

Getting help early is a key to successful treatment.

To get on top of depression and psychosis call
1300 73 22 11
or visit **www.getontop.org**

Example of Compass Postcards
