# Supplementary material for: Development and evaluation of a youth mental health community awareness campaign – The Compass Strategy
Source: BMC Public Health. 2006 Aug 22;6:215. doi: 10.1186/1471-2458-6-215 (PMC1564138; doi:10.1186/1471-2458-6-215)
Supplement: Additional File 4 — Example of Compass Poster [file 1471-2458-6-215-S4.pdf]

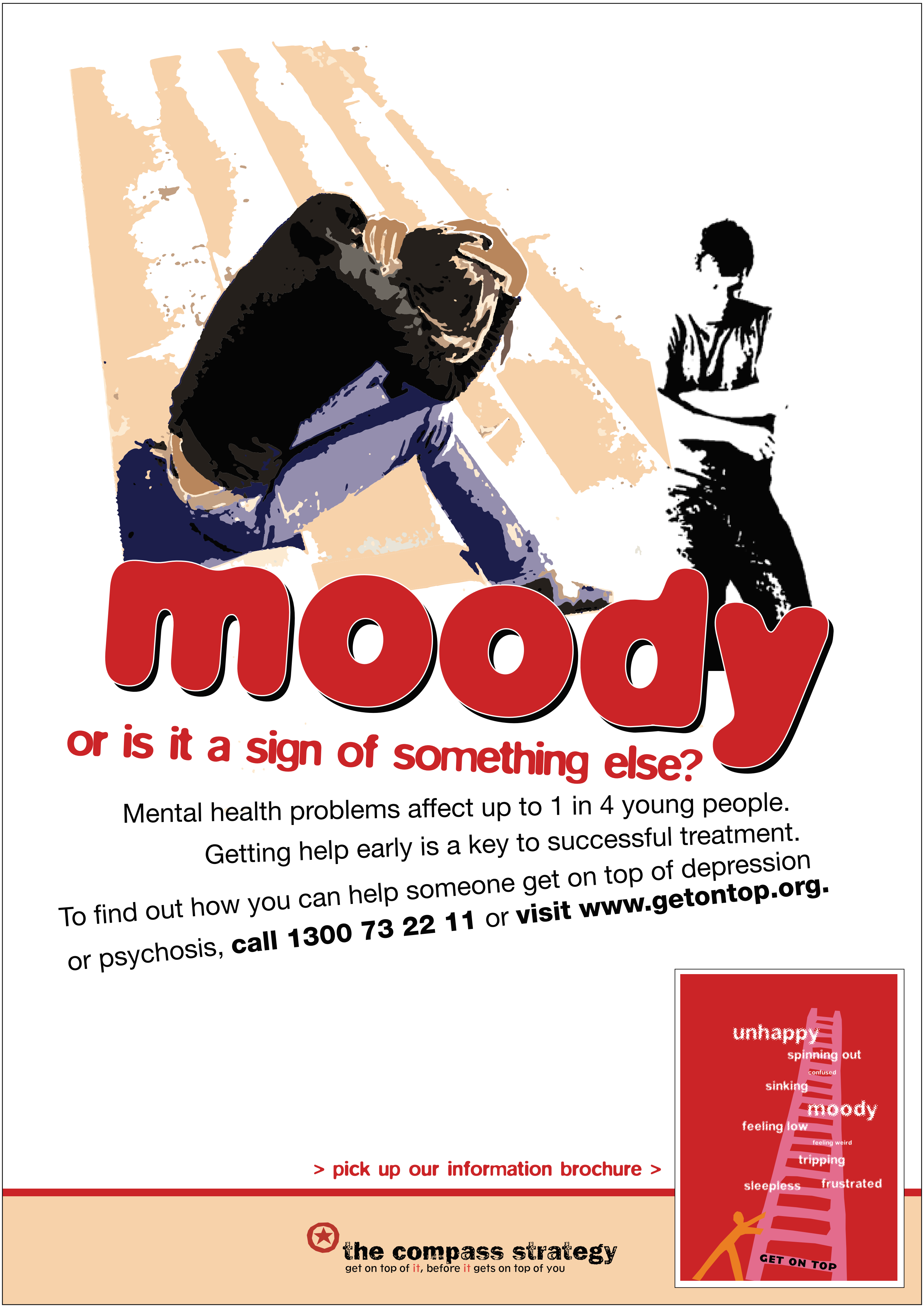

Example of Compass Poster
